# Ferritin, inflammation, and iron deficiency in acute heart failure: evidence from the EDIFICA cohort

**DOI:** 10.1007/s00392-024-02535-x

**Published:** 2024-09-19

**Authors:** Francisco Vasques-Nóvoa, Maria João Pimentel, Pedro Marques, Catarina Vale, Filipa Gomes, João Sérgio Neves, Isaac Barroso, João Tiago Guimarães, Paulo Bettencourt, Adelino F. Leite-Moreira, Roberto Roncon-Albuquerque, Jorge Almeida, João Pedro Ferreira, Fernando Friões

**Affiliations:** 1 Department of Internal Medicine, Centro Hospitalar Universitário de São João, Porto, Portugal; 2 Cardiovascular Research and Development Center (UnIC@RISE), Department of Surgery and Physiology, Faculty of Medicine of the University of Porto, Alameda Hernâni Monteiro, 4200-319 Porto, Portugal; 3 Instituto de Investigação e Inovação em Saúde (i3S), University of Porto, Porto, Portugal; 4 Department of Endocrinology, Centro Hospitalar Universitário de São João, Porto, Portugal; 5 Department of Clinical Pathology, Centro Hospitalar Universitário de São João, Porto, Portugal; 6 Université de Lorraine, Inserm, Centre d’Investigations Cliniques, Plurithématique 14-33, and Inserm U1116, CHRU Nancy, F-CRIN INI-CRCT (Cardiovascular and Renal Clinical Trialists), Nancy, France

**Keywords:** Acute heart failure, Ferritin, Inflammation, Iron deficiency

## Abstract

**Background:**

Ferritin is commonly used to evaluate iron stores and guide therapeutic decisions regarding intravenous iron supplementation. However, in the context of AHF, inflammation-driven upregulation of ferritin might disrupt its correlation with iron stores, restricting iron bioavailability and potentially amplifying the inflammatory response.

**Aim:**

This study aims to assess the clinical and prognostic associations of ferritin levels in an AHF cohort and to determine whether the prognostic value of ferritin is influenced by the presence of infection, inflammatory activation, and other markers of iron deficiency.

**Methods:**

The association between ferritin and clinical outcomes (180 days) in AHF was evaluated in a cohort of 526 patients from the EDIFICA registry.

**Results:**

The median ferritin plasma concentration at admission was 180 pg/mL. Patients with higher ferritin levels at admission were predominantly men, exhibiting a high prevalence of chronic kidney disease and alcohol consumption, and presenting with lower blood pressure and a higher incidence of clinical infection. Higher ferritin levels were associated with increased risk of the composite of heart failure hospitalization or cardiovascular death (Tertile 2: HR 1.75; 95% CI 1.10–2.79; *p* = 0.017; Tertile 3: HR 1.79; 95% CI 1.08–2.97; *p* = 0.025), independently of classical HF prognostic factors, inflammatory and iron-related markers. No significant associations were found between admission serum iron or transferrin saturation tertiles, iron status categories, or guideline-defined iron deficiency (ID) criteria and the primary composite outcome. However, at discharge, patients who met the criteria for defective iron utilization, low iron storage, or guideline-defined ID had a lower risk of the composite endpoint compared to those with normal iron utilization or who did not meet the guideline-defined ID criteria, respectively.

**Conclusions:**

Elevated ferritin levels are independently associated with poor prognosis in AHF. Low ferritin levels are associated with a favorable outcome and do not carry significant value in identifying ID in this population.

**Supplementary Information:**

The online version contains supplementary material available at https://doi.org/10.1007/s00392-024-02535-x.

## Introduction

Acute heart failure (AHF) stands as a leading cause of unplanned hospitalization and portends a poor prognosis [1]. Inflammatory activation is frequently observed in AHF patients, either due to infectious triggers leading to HF decompensation (e.g., respiratory infection) or as a result of exacerbation of HF alongside comorbid conditions (e.g., worsening of renal function) [2, 3].

Ferritin is a central regulator of intracellular iron levels and its plasma concentrations are widely used for assessing the body iron stores, particularly for establishing the diagnosis of iron deficiency (ID) and making treatment decisions regarding the administration of IV iron therapy [1, 4, 5]. However, in inflammatory conditions, ferritin levels are highly upregulated independently of iron status [6]. Consequently, within the context of AHF, high-ferritin levels might primarily indicate the presence of inflammatory activation rather than excluding ID. Despite this theoretical background, there is insufficient evidence on the relationship between ferritin, markers of ID and clinical outcomes in AHF.

## Aims

To evaluate the clinical and prognostic associations of ferritin levels (both at hospital admission and discharge) in an AHF cohort. Assess whether the prognostic value of ferritin is influenced by the presence of infection, inflammatory activation, and biomarkers of iron deficiency. Lastly, we aim to discuss the potential implications of the use of ferritin for the criteria of ID and iron replacement therapy in AHF.

## Methods

The EDIFICA registry (“Estratificação de Doentes com InsuFIciência Cardíaca Aguda”) is a single-center, prospective cohort study, conducted during a 21-month period at the Internal Medicine Department of Centro Hospitalar Universitário São João, Porto, Portugal [2, 7]. Patients admitted with the primary diagnosis of AHF were eligible for registry inclusion. Patients with acute coronary syndromes and those whose symptoms were explained by conditions other than HF were excluded from this registry. All patients underwent detailed clinical history, physical examination, and venous blood sample analysis at admission and discharge. An echocardiogram was performed within 72 h after admission. The investigation conformed with the principles outlined in the Declaration of Helsinki and was approved by the local ethics committee. Admission (*N* = 526) and discharge (*N* = 396) ferritin levels were evaluated using an immunoturbidimetric assay (OSR61203; Beckman Coulter).

Categorical variables are described as frequencies (proportions), and continuous variables are expressed as means ± standard deviation or median (25th and 75th percentiles), depending on their distributions. Comparisons of demographic, clinical, and biological parameters among tertiles of ferritin levels, iron status [8] and guideline-defined iron deficiency criteria [1] were analyzed using analysis of variance (ANOVA) for continuous normally distributed variables, the analogous non-parametric Kruskal–Wallis test for continuous non-normally distributed variables, and Chi-squared test (*χ*^2^) for categorical variables. The primary endpoint was a composite of heart failure hospitalization (HFH) or cardiovascular death (CVD). Cox proportional hazards regression models were used to assess the association between ferritin levels and the clinical endpoints, adjusting for sex, age, atrial fibrillation, NYHA class at admission, eGFR (CKD-EPI formula), clinical infection and circulating levels of B-type natriuretic peptide (BNP, log_2_), transferrin saturation (TSAT, log_2_), hemoglobin, albumin, total cholesterol, and C-reactive protein (CRP, log_2_). All statistical analyses were performed with Stata^®^ (StataCorp. 2021. Stata Statistical Software: Release 18.0. College Station, TX: StataCorp LLC). A 2-sided value of p<0.05 was considered statistically significant. 

## Results

The median age was 78 (IQR 71–84) years, and 227 (43%) patients were men. Median ejection fraction was 41%, 228 (43%) patients had an ejection fraction ≤ 40% and 40% of the patients had HF of ischemic etiology. Median ferritin plasma concentration was 180 pg/mL (percentile_25-75_ 74–312 pg/mL) at admission and 197 pg/mL (percentile_25-75_ 108–346 pg/mL) at discharge.

Table 1 presents patient characteristics, demographic data, and analytical parameters by tertiles of ferritin levels at admission. Patients in the highest tertile of ferritin at admission (Tertile 3: 421 [312–611] pg/mL) were predominantly men, with a high prevalence of chronic kidney disease and alcohol consumption and presenting with lower blood pressure and a high incidence of clinical infection. On the other hand, patients with lower ferritin levels (Tertile 1: 57 [36–74] pg/mL) used beta-blockers and loop diuretics less frequently and had higher rate of atrial fibrillation. No differences were observed between ferritin tertiles regarding age, HF disease-modifying treatment (e.g., ACEi, ARB or MRAs), circulating BNP and troponin or classical cardiovascular risk factors such as anemia, diabetes and hypertension. Increased ferritin correlated with higher mean corpuscular volume, TSAT, urea, C-reactive protein, and lower red blood cell distribution width and transferrin concentration. These associations remained consistent when discharge ferritin tertiles were analyzed (Table S1).

**Table 1:** Baseline characteristics by admission ferritin tertiles

Characteristic	Tertile 1 (7.3–102.2 ng/mL) *N* = 176	Tertile 2 (105.5–250.1 ng/mL) *N* = 175	Tertile 3 (252.9–8669.4 ng/mL) *N* = 175	*p*-value1
Age (years)	77 (71, 84)	78 (72, 84)	80 (71, 85)	0.6
Male sex	54 (30.7%)	78 (44.6%)	95 (54.3%)	** < 0.001**
BMI (Kg/m^2^)	25.6 ± 4.6	25.6 ± 4.6	24.8 ± 4.1	0.13
Previous medical history				
Dyslipidemia	79 (54.9)	110 (72.4)	71 (46.7)	** < 0.001**
Chronic kidney disease	25 (15.1)	41 (23.8)	56 (34.6)	** < 0.001**
Alcohol consumption	54 (31.0)	69 (39.9)	83 (47.7)	**0.006**
Atrial fibrillation	90 (51.4)	67 (38.3)	73 (42.0)	**0.038**
Anemia	81 (48.5)	72 (41.9)	89 (52.7)	0.1
Diabetes mellitus	65 (37.6)	81 (46.6)	67 (38.7)	0.2
Myocardial infarction	45 (25.9)	59 (34.1)	58 (33.3)	0.2
Hypertension	126 (73.7)	137 (80.6)	121 (74.2)	0.3
COPD	25 (17.6)	33 (22.9)	35 (24.0)	0.4
Previous medication				
Beta-blocker	68 (38.9)	92 (53.5)	96 (56.1)	**0.002**
ACEi or ARB	100 (57.1)	114 (66.3)	108 (63.2)	0.2
MRA	25 (14.3)	23 (13.4)	28 (16.4)	0.8
Loop diuretics	115 (65.7)	138 (79.3)	127 (73.8)	**0.016**
Statins	81 (46.3)	99 (57.6)	70 (40.9)	**0.007**
Clinical presentation and hospital stay				
Systolic blood pressure (mmHg)	134 (116, 155)	133 (117, 154)	123 (106, 145)	**0.002**
Diastolic blood pressure (mmHg)	76 (66, 90)	75 (65, 90)	70 (59, 80)	**0.001**
Heart rate (bpm)	86 (75, 102)	85 (71, 98)	85 (71, 101)	0.4
Clinical infection	57 (33.3)	74 (42.8)	107 (62.2)	** < 0.001**
Length of stay (days)	8.0 (6.0, 13.0)	7.0 (5.0, 10.5)	8.0 (6.0, 13.0)	**0.007**
Admission analytical evaluation				
Anemia				
None	72 (40.9)	57 (32.6)	58 (33.1)	0.1
Mild	35 (19.9)	40 (22.9)	28 (16.0)	
Moderate/severe	69 (39.2)	78 (44.6)	89 (50.9)	
Hemoglobin (g/dL)	11.8 (10.2, 13.4)	11.5 (10.6, 12.7)	11.4 (10.0, 13.0)	0.6
MCV (fL)	87.0 (81.2, 91.7)	91.5 (87.8, 96.2)	92.1 (88.2, 95.5)	** < 0.001**
RDW-CV (%)	15.5 (14.4, 17.1)	14.7 (14.1, 16.0)	14.6 (13.8, 16.0)	** < 0.001**
AST (U/L)	27.0 (21.0, 35.0)	25.5 (19.0, 35.0)	28.0 (21.0, 43.0)	0.1
Albumin (g/L)	35.5 (32.8, 38.2)	35.4 (32.2, 38.5)	35.1 (31.7, 38.0)	0.6
Total cholesterol (mg/dL)	147.5 (123.0, 185.0)	151.0 (119.0, 185.0)	154.0 (127.5, 184.0)	0.5
HbA1c (%)	6.3 (5.8, 7.1)	6.1 (5.7, 6.8)	6.2 (5.6, 7.0)	0.08
CKD-EPI eGFR (mL/min/1.73 m^2^)	44.1 (30.1, 56.3)	40.5 (29.9, 56.3)	40.7 (26.9, 56.9)	0.6
Urea (mg/dL)	60 (43, 91)	66 (51, 98)	74 (51, 106)	**0.019**
Iron (mg/mL)	34.5 (22.3, 48.0)	43.0 (28.0, 63.0)	39.0 (29.0, 57.0)	** < 0.001**
Transferrin (mg/dL)	296.0 (261.5, 334.5)	231.0 (205.0, 266.0)	197.0 (173.0, 240.5)	** < 0.001**
Transferrin saturation (%)	7.8 (5.3, 12.7)	13.1 (8.5, 19.7)	14.4 (10.6, 19.2)	** < 0.001**
C-reactive protein (mg/L)	12.8 (6.1, 24.7)	20.8 (10.1, 58.9)	39.3 (14.9, 110.5)	** < 0.001**
Troponin I (ng/mL)	0.1 (0.0, 0.2)	0.0 (0.0, 0.1)	0.1 (0.0, 0.2)	0.07
BNP (pg/mL)	1328.9 (761.9, 2676.7)	1169.8 (666.2, 2496.0)	1483.0 (678.0, 2666.8)	0.5
Echocardiographic evaluation				
Ejection fraction (%)	43.0 (23.0, 57.0)	37.0 (22.8, 55.0)	42.5 (26.0, 58.0)	0.3
LAVI (mL/m^2^)	51.6 (40.2, 66.1)	49.1 (40.1, 61.4)	45.5 (34.6, 59.4)	**0.018**
LVMI (g/m^2^)	129.9 (108.5, 160.5)	136.0 (112.2, 162.2)	131.2 (111.2, 152.1)	0.5
sPAP (mmHg)	45.9 (38.1, 54.0)	47.3 (39.4, 57.2)	47.2 (37.8, 59.4)	0.5
Global circumferential strain (%)	– 8.1 (– 11.5, – 6.0)	– 9.6 (– 14.3, – 6.2)	– 9.4 (– 12.4, – 6.5)	0.2
Global longitudinal strain (%)	– 9.5 (– 13.5, – 7.0)	– 11.2 (– 15.0, – 7.6)	– 10.0 (– 15.2, – 6.4)	0.4

The bold indicates the p<0.05

*BMI* Body mass index; *COPD* chronic obstructive pulmonary disease; *MCV* mean corpuscular volume; *ACEi* angiotensin-converting enzyme inhibitor; *ARB* angiotensin receptor blocker; *MRA* mineralocorticoid receptor antagonist; *RDW-CV* cell distribution width-CV; AST aspartate aminotransferase; *HbA1c* A1c; *CKD-EPI eGFR* CKD-EPI estimated glomerular filtration rate; *BNP* brain natriuretic peptide; *LAVI* left atrial Volume index; *LVMI* left ventricular mass index; *sPAP* systolic pulmonary arterial pressure

**Fig. 1:**
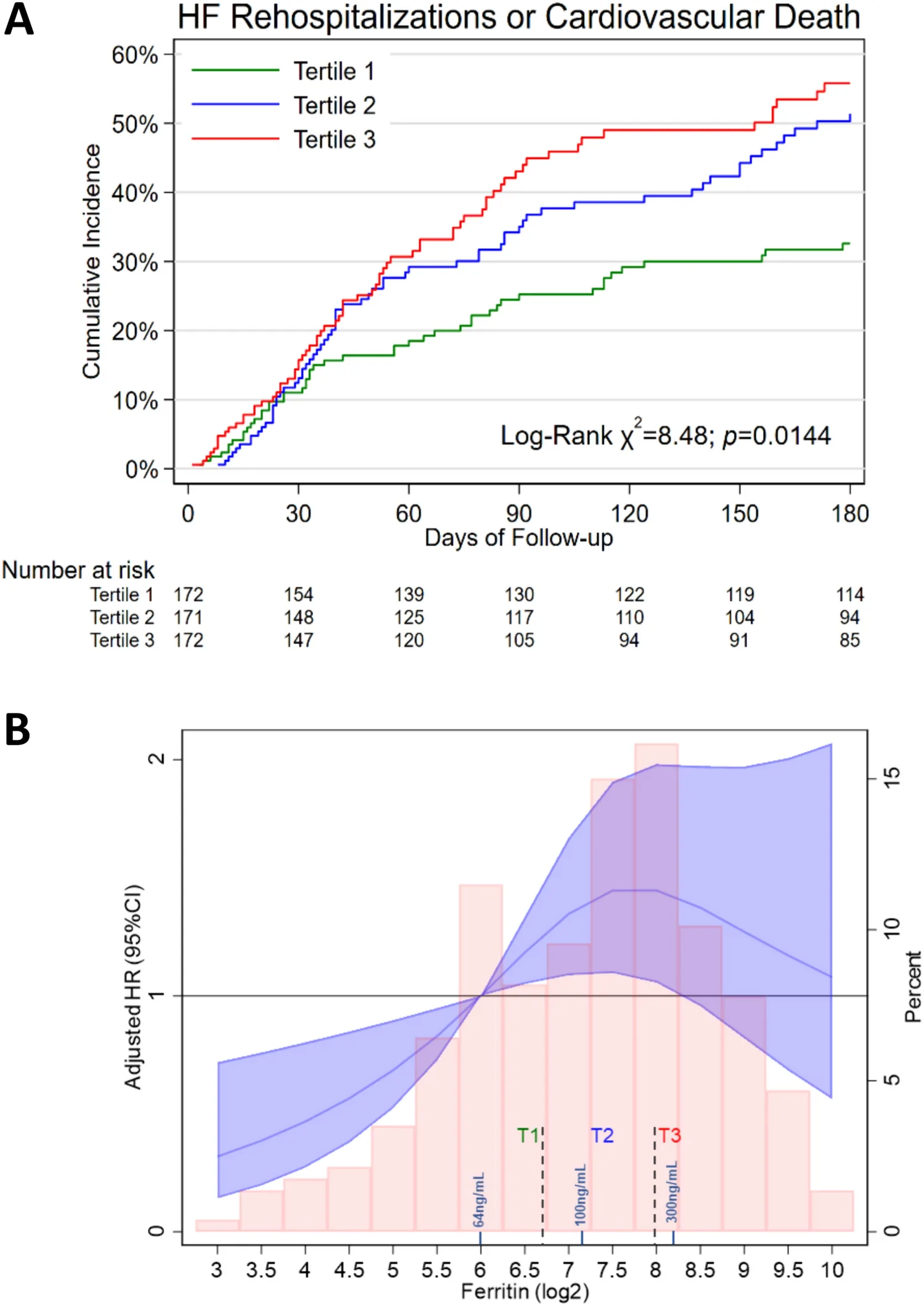
Admission ferritin levels and acute heart failure outcomes. **A** Kaplan–Meier analysis for 180-day composite endpoint of HF hospitalization or cardiovascular death, stratified by tertiles of admission ferritin. **B** Spline curve for the composite endpoint of HF hospitalization and cardiovascular death across the range of admission ferritin values (log_2_). The hazard ratio is depicted by a green line, accompanied by overlaid 95% confidence intervals (green shadow) alongside an admission ferritin histogram (red bars)

Table 2 shows the association between admission ferritin levels and clinical outcomes. Higher ferritin levels (Tertiles 2 and 3) were independently associated with increased risk of the composite of HFH or cardiovascular death (Tertile 2: HR 1.75; 95% CI 1.10–2.79; *p* = 0.017; Tertile 3: HR 1.79; 95% CI 1.08–2.97; *p* = 0.025) (Fig. 1). This association remained consistent across all subgroups, including among patients presenting with clinical infection (Table 3). Similar, though less strong, associations were observed when analyzing discharge ferritin tertiles (Table S2 and Fig. S1) or applying guideline-defined ID criteria ferritin cutoffs (Table S3 and Fig. S2). Patient characteristics based on admission and discharge iron status and guideline-defined ID criteria ferritin cutoffs are additionally displayed in the supplemental data (Tables S4 and S5).

**Table 2:** Adverse clinical outcomes by admission ferritin tertiles

Outcomes^1^	*N* (%)	Unadjusted	*p-*value	Adjusted^2^	*p-*value
HF hospitalization or cardiovascular death					
Tertile 1 [7.3–102.2 ng/mL]	47/176 (26.7)	Reference		Reference	
Tertile 2 [105.5–250.1 ng/mL]	67/175 (38.3)	1.52 (1.04–2.21)	**0.027**	1.75 (1.10–2.79)	**0.017**
Tertile 3 [252.9–8669.4 ng/mL]	71/175 (40.6)	1.69 (1.17–2.44)	**0.005**	1.79 (1.08–2.97)	**0.025**
HF hospitalization					
Tertile 1 [7.3–102.2 ng/mL]	34/176 (19.3)	Reference		Reference	
Tertile 2 [105.5–250.1 ng/mL]	55/175 (31.4)	1.74 (1.14–2.68)	**0.011**	1.70 (0.99–2.89)	0.053
Tertile 3 [252.9–8669.4 ng/mL]	41/175 (23.4)	1.37 (0.87–2.16)	0.2	1.36 (0.74–2.49)	0.3
Cardiovascular death					
Tertile 1 [7.3–102.2 ng/mL]	23/176 (13.1)	Reference		Reference	
Tertile 2 [105.5–250.1 ng/mL]	25/175 (14.3)	1.05 (0.60–1.86)	0.9	1.55 (0.78–3.08)	0.2
Tertile 3 [252.9–8669.4 ng/mL]	30/175 (21.7)	1.73 (1.03–2.91)	**0.036**	1.91 (0.88–4.12)	0.099
All-cause hospitalization or death					
Tertile 1 [7.3–102.2 ng/mL]	79/176 (44.9)	Reference		Reference	
Tertile 2 [105.5–250.1 ng/mL]	188/175 (50.3)	1.19 (0.87–1.69)	0.3	1.29 (0.89–1.90)	0.2
Tertile 3 [252.9–8669.4 ng/mL]	100/175 (57.1)	1.45 (1.07–1.95)	**0.014**	1.52 (1.01–2.29)	**0.047**
All-cause hospitalization					
Tertile 1 [7.3–102.2 ng/mL]	59/176 (33.5)	Reference		Reference	
Tertile 2 [105.5–250.1 ng/mL]	74/175 (42.3)	1.34 (0.95–1.90)	0.090	1.34 (0.87–2.06)	0.2
Tertile 3 [252.9–8669.4 ng/mL]	69/175 (39.3)	1.35 (0.95–1.92)	0.091	1.30 (0.80–2.10)	0.3
All-cause death					
Tertile 1 [7.3–102.2 ng/mL]	44/176 (25.0)	Reference		Reference	
Tertile 2 [105.5–250.1 ng/mL]	41/175 (23.4)	0.91 (0.59–1.39)	0.7	0.99 (0.59–1.65)	0.5
Tertile 3 [252.9–8669.4 ng/mL]	55/175 (31.4)	1.34 (0.90–1.99)	0.2	1.19 (0.67–2.10)	0.5

The bold indicates the p<0.05

^1^Hazard ratios (HR) are presented with their 95% confidence interval

^2^Adjusted for sex, age, atrial fibrillation, NYHA class, creatinine_log2_, BNP_log2_, albumin, haemoglobin, TSAT_log2_, total cholesterol, CRP_log2_ and clinical infection

**Table 3:** Subgroup analysis of the association of ferritin tertiles with primary composite endpoint

Outcome	Tertile 1 (7.3–102.2 ng/mL) *N* = 176	Tertile 2 (105.5–250.1 ng/mL) *N* = 175	Tertile 3 (252.9–8669.4 ng/mL) *N* = 175	Interaction *p*-value
Age (years)				
< 78	Reference	1.88 (1.06–3.35)	1.95 (1.08–3.50)	0.6
≥ 78	Reference	1.25 (0.76–2.04)	1.42 (0.89–2.29)	
Sex				
Female	Reference	1.83 (1.12–3.01)	1.85 (1.11–3.09)	0.4
Male	Reference	1.07 (0.61–1.91)	1.31 (0.76–2.27)	
Ejection fraction (%)				
< 40%	Reference	1.12 (0.65–1.95)	1.70 (0.99–2.91)	0.2
≥ 40%	Reference	1.94 (1.17–3.23)	1.67 (1.01–2.77)	
Anemia				
No	Reference	1.78 (0.88–3.65)	1.88 (0.93–3.80)	0.8
Yes	Reference	1.36 (0.88–2.11)	1.57 (1.02–2.41)	
eGFR (mL/min/1.73 m^2^)				
< 42	Reference	0.98 (0.61–1.57)	1.13 (0.71–1.80)	0.06
≥ 42	Reference	2.39 (1.29–4.44)	2.60 (1.39–4.72)	
Iron (mg/mL)				
< 38.5	Reference	1.67 (1.03–2.73)	1.64 (1.01–2.66)	0.8
≥ 38.5	Reference	1.46 (0.80–2.65)	1.82 (1.01–3.28)	
Transferrin saturation (%)				
< 11.8%	Reference	1.67 (1.02–2.72)	1.37 (0.78–2.41)	0.4
≥ 11.8%	Reference	1.35 (0.69–2.62)	1.81 (0.96–3.43)	
Infection				
No	Reference	1.29 (0.77–2.13)	1.92 (1.14–3.22)	0.2
Yes	Reference	1.63 (0.92–2.88)	1.33 (0.76–2.32)	
C-reactive protein (mg/L)				
< 21	Reference	1.27 (0.76–2.12)	1.70 (0.99–2.94)	0.6
≥ 21	Reference	1.52 (0.84–2.74)	1.39 (0.79–2.47)	

No significant associations were found between admission serum iron or TSAT tertiles, iron status categories, or guideline-defined iron deficiency criteria and the primary composite outcome (Table 4). Contrastingly, at discharge, intermediate tertiles of serum iron (Tertile 2: 53 [42–68] μg/mL) and TSAT (Tertile 2: 15.5 [11.8–20.2] %) demonstrated a decreased risk of the primary composite endpoint when compared to those in the highest tertile (Tertile 3), which encompasses the ‘normal range’ (Table S6). Accordingly, patients who met the criteria at discharge for defective iron utilization and low iron storage, or the guideline-defined ID criteria, had a lower risk of the primary composite endpoint compared to those with normal iron utilization and those who did not meet the guideline-defined ID criteria, respectively (Table S6).

**Table 4:** Primary composite endpoint by admission serum iron, transferrin saturation, iron status, anemia and guideline-defined ID criteria

Primary composite endpoint^1^					
Admission	*N* (%)	Unadjusted	*p*-value	Adjusted	*p-*value
Serum iron^2,3^					
Tertile 1 [4–32 mg/mL]	63/167 (37.7)	1.14 (0.84–1.56)	0.4	0.71 (0.46–1.10)	0.2
Tertile 2 [33–46 mg/mL]	52/168 (31.0)	0.91 (0.66–1.27)	0.6	0.78 (0.51–1.18)	0.2
Tertile 3 [47–314 mg/mL]	57/164 (34.8)	Reference		Reference	
Transferrin saturation^2^					
Tertile 1 [1.73–8.51%]	56/166 (33.7)	0.94 (0.65–1.34)	0.7	0.70 (0.46–1.06)	0.095
Tertile 2 [8.52–15.16%]	55/166 (33.1)	0.92 (0.64–1.33)	0.7	1.03 (0.58–1.31)	0.5
Tertile 3 [15.24–93.42%]	61/166 (36.8)	Reference		Reference	
Iron status^2^					
Normal iron status	45/116 (38.8)	Reference		Reference	
Defective iron Utilization	54/180 (30.0)	0.73 (0.49–1.08)	0.1	0.66 (0.40–1.08)	0.3
Low iron storage	86/230 (37.2)	0.96 (0.67–1.38)	0.8	1.01 (0.64–1.62)	0.9
Anemia^3^					
None	50/187 (26.7)	Reference		Reference	
Mild	42/103 (40.8)	1.68 (1.11–2.53)	**0.014**	1.65 (1.03–2.63)	**0.034**
Moderate/severe	93/236 (39.4)	1.65 (1.17–2.34)	**0.004**	1.33 (0.88–2.03)	0.2
Guideline ID criteria^2^					
Yes	108/316 (34.2)	Reference		Reference	
No	77/210 (36.7)	1.11 (0.82–1.49)	0.5	0.98 (0.67–1.40)	0.9

The bold indicates the p<0.05

^1^Hazard ratios (HR) are presented with their 95% confidence interval

^2^Adjusted for adjusted for sex, age, atrial fibrillation, NYHA class, creatinine_log2_, BNP_log2_, albumin, haemoglobin, total cholesterol, CRP_log2_ and clinical infection

^3^Serum iron conversion factor from mg/mL to μmol/μL - divide by 5.59

^4^Adjusted for sex, age, atrial fibrillation, NYHA class, creatinine_log2_, BNP_log2_, albumin, TSAT_log2_, total cholesterol, CRP_log2_ and clinical infection

Comparison of multiple established iron deficiency-related cutoffs revealed increased circulating levels of ferritin, transferrin saturation, and serum iron at discharge, alongside a decreased prevalence of anemia, compared with admission (Table S7).

## Conclusion

In this study, we show that higher ferritin levels are associated with worse clinical outcomes, independently of key HF prognostic factors, iron-related factors and inflammatory markers, suggesting potential ferritin-dependent mechanisms of HF decompensation [9, 10]. Importantly, our data also indicate that in the inflammatory context of AHF, measuring ferritin, serum iron, or transferrin saturation may not reliably assess iron status, challenging the validity of current guideline-defined criteria for identifying ID in this clinical setting.

Inflammation plays a central role in the pathogenesis and prognosis of AHF, as evidenced by the high prognostic value of inflammatory markers [2]. Ferritin expression is markedly upregulated by inflammatory signaling and is associated with adverse outcomes across a broad range of cardiovascular, infectious, immunological, and malignant disorders [6, 11, 12]. Interestingly, in inflammatory states, particularly during infection, ferritin upregulation has been perceived as protective, as it restricts iron availability to pathogens and attenuates iron-mediated oxidative stress [6]. However, experimental data suggests a potential dual role of ferritin, as it can also apparently function as a cytokine, increasing inflammatory transcriptional activity [13]. Furthermore, within the context of AHF, the impairment of iron bioavailability can negatively impact on myocardial bioenergetics and function [14].

Importantly, categorization by iron status and guideline-defined ID criteria at discharge identified patients at lower, instead of higher, risk of clinical events. These results suggest that AFFIRM-AHF [15] and IRONMAN [16] trials, by selecting a low-ferritin AHF population, may have targeted a low-inflammation population including patients potentially lacking ID. Simultaneously, these trials may have excluded patients with ID and high inflammatory activity, who could have potentially benefited from the treatment outside the hyperinflammatory phase. While it is seems reasonable to exclude patients with a confirmed or suspected active infection from IV iron therapy [17, 18], it is important to acknowledge that AHF itself represents an inflammatory condition and high-ferritin patients may still benefit from IV supplementation. Supporting this hypothesis, a secondary analysis from the IRONMAN trial recently revealed that patients with higher ferritin (i.e., > 100 ng/mL) had worse outcome but showed a greater response to IV iron therapy [19].

Transferrin saturation or serum iron concentration were recently shown to outperform guideline-defined criteria in predicting HF-related mortality and may serve as viable alternatives [12, 19, 20]. However, caution should be exercised when applying cutoffs derived from chronic HF cohorts to the inflammatory setting of AHF, as our analysis suggest pre-discharge levels below these cutoffs (e.g. TSAT < 20%) may offer some protection. In addition, the negative correlation between inflammation and transferrin may complicates the use classical ID criteria in high-ferritin states, such as AHF [21]. In this regard, soluble transferrin receptor, which has been shown to accurately reflect iron stores and independently predict HF outcomes [22, 23], could potentially improve the assessment of iron status in AHF, but further studies are needed to confirm its utility in this setting.

In conclusion, in patients with AHF, elevated ferritin levels are associated with poor prognosis, independently of classical HF prognosticators and inflammatory markers. Low ferritin levels are associated with a favorable outcome and may not hold significant value in identifying ID in this population.

## Supplementary Information

Below is the link to the electronic supplementary material.

Supplementary file1 (DOCX 146 KB)
