# Supplementary material for: Ferritin, inflammation, and iron deficiency in acute heart failure: evidence from the EDIFICA cohort
Source: Clin Res Cardiol. 2024 Sep 19;115(11):1889–96. doi: 10.1007/s00392-024-02535-x (PMC13658546; doi:10.1007/s00392-024-02535-x)
Supplement: Supplementary file 1 [file 392_2024_2535_MOESM1_ESM.docx]

**SUPPLEMENTAL FIGURE LEGENDS**

***Supplemental Figure 1.* Discharge ferritin levels and acute heart failure outcomes**

**(A)** Kaplan-Meier analysis for 180-day composite endpoint of HF hospitalization or cardiovascular death, stratified by tertiles of discharge ferritin. **(B)** Spline curve for the composite endpoint of HF hospitalization and cardiovascular death across the range of discharge ferritin values (log2). The hazard ratio is depicted by a green line, accompanied by overlaid 95% confidence intervals (green shadow) alongside an admission ferritin histogram (red bars).

***Supplemental Figure 2.* Iron deficiency guideline criteria ferritin cutoffs and acute heart failure outcomes**

Kaplan-Meier analysis for 180-day composite endpoint of HF hospitalization or cardiovascular death, stratified by admission **(A)** and discharge **(B)** ferritin categories (<100, 100-299 and >300ng/mL).

**Table S1.** Baseline Characteristics by Discharge Ferritin Tertiles

| **Characteristic** | | | **Tertile 1**  ***(0.2-134.8 ng/mL)***  N = 132 | | | **Tertile 2**  ***(135.0-286.4 ng/mL)***  N = 132 | | | **Tertile 3**  ***(288.0-*** ***2545.2 ng/mL)***  N = 132 | | | **p-value** | |  |  |
| --- | --- | --- | --- | --- | --- | --- | --- | --- | --- | --- | --- | --- | --- | --- | --- |
| **Age** (years) | | | 76.5 (70.5, 84.0) | | | 77.0 (72.0, 84.0) | | | 80.0 (69.0, 85.0) | | | 0.60 | |  |  |
| **Male sex** | | | 39 (29.5) | | | 71 (53.8) | | | 69 (52.3) | | | **<0.001** | |  |  |
| **BMI** (Kg/m^2^) | | | 25.8 ± 4.5 | | | 25.5 ± 4.4 | | | 25.1 ± 4.5 | | | 0.54 | |  |  |
|  | | |  | | |  | | |  | | |  | |  |  |
| **PREVIOUS MEDICAL HISTORY** |  | | |  | | |  | | |  | | |  |  |  |
| **Dyslipidemia** | | | 68 (60.7) | | | 71 (61.2) | | | 62 (53.4) | | | 0.41 | |  |  |
| **Chronic Kidney Disease** | | | 22 (17.1) | | | 30 (24.0) | | | 39 (31.0) | | | **0.034** | |  |  |
| **Alcohol Consumption** | | | 39 (30.0) | | | 52 (39.7) | | | 65 (49.6) | | | **0.005** | |  |  |
| **Atrial Fibrillation** | | | 65 (49.6) | | | 54 (40.9) | | | 48 (36.4) | | | 0.09 | |  |  |
| **Anemia** | | | 59 (45.4) | | | 58 (46.0) | | | 69 (53.5) | | | 0.35 | |  |  |
| **Diabetes Mellitus** | | | 56 (43.1) | | | 55 (42.0) | | | 55 (42.0) | | | 0.98 | |  |  |
| **Myocardial Infarction** | | | 39 (29.8) | | | 42 (32.3) | | | 44 (33.6) | | | 0.80 | |  |  |
| **Hypertension** | | | 101(77.1) | | | 101(77.7) | | | 93 (73.8) | | | 0.74 | |  |  |
| **COPD** | | | 21 (19.1) | | | 23 (20.7) | | | 22 (19.5) | | | 0.95 | |  |  |
|  | | |  | | |  | | |  | | |  | |  |  |
| **PREVIOUS MEDICATION** | | |  | | |  | | |  | | |  | |  |  |
| **Beta-blocker** | | | 57 (43.2) | | | 72 (55.4) | | | 66 (51.2) | | | 0.13 | |  |  |
| **ACEi or ARB** | | | 53 (40.2) | | | 55 (42.3) | | | 62 (48.1) | | | 0.41 | |  |  |
| **MRA** | | | 21 (15.9) | | | 15 (11.5) | | | 24 (18.6) | | | 0.28 | |  |  |
| **Loop diuretics** | | | 84 (63.6) | | | 105(80.2) | | | 94 (71.8) | | | **0.012** | |  |  |
| **Statins** | | | 67 (50.8) | | | 63 (48.5) | | | 57 (44.2) | | | 0.56 | |  |  |
|  | | |  | | |  | | |  | | |  | |  |  |
| **CLINICAL PRESENTATION AND HOSPITAL STAY** | | |  | | |  | | |  | | |  | |  |  |
| **Systolic blood pressure** (mmHg) | | | 114.0 (102.0, 130.0) | | | 112.0 (100.0, 125.0) | | | 110.0 (100.0, 124.0) | | | 0.27 | |  |  |
| **Diastolic blood pressure** (mmHg) | | | 64.0 (53.0, 72.0) | | | 63.5 (57.0, 75.0) | | | 65.0 (56.0, 70.0) | | | 0.97 | |  |  |
| **Heart rate** (bpm) | | | 71.5 (65.0, 82.0) | | | 72.0 (65.0, 81.0) | | | 74.0 (67.0, 82.0) | | | 0.77 | |  |  |
| **Clinical infection** | | | 47 (36.2) | | | 51 (39.2) | | | 81 (62.3) | | | **<0.001** | |  |  |
| **Length of Stay** (days) | | | 7.0(5.0, 11.0) | | | 7.0(5.0, 11.0) | | | 8.0(6.0, 14.0) | | | **0.022** | |  |  |
|  | | |  | | |  | | |  | | |  | |  |  |
| **ADMISSION ANALYTICAL EVALUATION** |  | | |  | | |  | | |  | | |  |  |  |
| **Anemia**  *None*  *Mild*  *Moderate/Severe* | | | 54 (40.9)  30 (22.7)  48 (36.4) | | | 47 (35.6)  22 (16.7)  63 (47.7) | | | 52 (39.4)  25 (18.9)  55 (41.7) | | | 0.4 | |  |  |
| **MCV** (fL) | | | 87.2 (83.1, 91.3) | | | 91.1 (87.1, 94.7) | | | 91.8 (88.2, 94.5) | | | **<0.001** | |  |  |
| **AST** (U/L) | | | 24.0 (17.0, 31.0) | | | 24.0 (20.0, 33.5) | | | 28.5 (21.0, 41.0) | | | **0.008** | |  |  |
| **ALT** (U/L) | | | 18.5 (14.0, 31.0) | | | 23.0 (15.0, 30.0) | | | 28.5 (17.0, 47.0) | | | **<0.001** | |  |  |
| **Albumin** (g/L) | | | 35.8 (32.3, 38.9) | | | 34.8 (31.2, 38.2) | | | 34.8 (30.9, 37.7) | | | 0.15 | |  |  |
| **Total cholesterol** (mg/dL) | | | 147.0 (119.0, 175.0) | | | 143.0 (124.0, 174.0) | | | 154.5 (131.0, 183.5) | | | 0.12 | |  |  |
| **LDL cholesterol** (mg/dL) | | | 91.0 (73.0, 116.0) | | | 97.5 (74.0, 118.0) | | | 101.5 (85.0, 126.0) | | | **0.05** | |  |  |
| **HbA1c** () | | | 6.4 (5.9, 7.5) | | | 6.0 (5.8, 6.7) | | | 6.3 (5.7, 7.2) | | | **0.007** | |  |  |
| **Creatinine** (mg/dL) | | | 1.2 (1.0, 1.6) | | | 1.4 (1.1, 1.9) | | | 1.3 (1.0, 1.8) | | | **0.007** | |  |  |
| **Cystatin C** (mg/dL) | | | 1.3 (1.1, 1.7) | | | 1.6 (1.1, 2.1) | | | 1.5 (1.1, 2.1) | | | **0.013** | |  |  |
| **CKD-EPI eGFR** (mL/min/1.73m^2^) | | | 46.8 (35.1, 59.7) | | | 42.2 (29.7, 54.2) | | | 43.6 (29.5, 60.5) | | | 0.13 | |  |  |
| **Urea** (mg/dL) | | | 61.0 (47.0, 89.0) | | | 74.0 (52.0, 102.0) | | | 76.0 (51.0, 114.0) | | | **0.021** | |  |  |
| **Iron** (μg/mL) | | | 46.0 (36.0, 65.5) | | | 56.3 (37.5, 78.0) | | | 59.0 (40.0, 93.5) | | | **0.012** | |  |  |
| **Transferrin** (mg/dL) | | | 294.8 (253.0, 339.0) | | | 236.5 (215.8, 271.8) | | | 209.5 (180.0, 247.0) | | | **<0.001** | |  |  |
| **Transferrin Saturation** () | | | 11.8 (8.6, 15.9) | | | 16.4 (10.9, 22.4) | | | 19.9 (13.5, 30.3) | | | **<0.001** | |  |  |
| **C-reactive protein** (mg/L) | | | 8.9(4.0, 17.0) | | | 12.5 (7.5, 25.4) | | | 17.1 (7.5, 40.0) | | | **<0.001** | |  |  |
| **Interleukin-6** (pg/mL) | | | 14.7 (6.5, 23.4) | | | 12.2 (7.7, 33.2) | | | 15.9 (9.8, 20.8) | | | 0.82 | |  |  |
| **Troponin I** (ng/mL) | | | 0.0 (0.0, 0.1) | | | 0.0 (0.0, 0.1) | | | 0.1 (0.0, 0.1) | | | 0.09 | |  |  |
| **BNP** (pg/mL) | | | 612.1 (252.0, 1556.6) | | | 844.4 (355.8, 1595.0) | | | 727.1 (258.1, 1383.6) | | | 0.33 | |  |  |
|  | |  | | |  | | |  | | |  | | | |  |
| **ECHOCARDIOGRAPHIC EVALUATION** | | |  | | |  | | |  | | |  | |  |  |
| **Ejection Fraction** () | | | 43.3 (23.0, 57.5) | | | 34.5 (23.0, 54.0) | | | 43.5 (27.5, 58.0) | | | 0.11 | |  |  |
| **LAVI** (mL/m^2^) | | | 51.6 (38.6, 64.1) | | | 50.6 (40.3, 62.3) | | | 43.4 (33.1, 53.3) | | | **0.004** | |  |  |
| **LVMI** (g/m^2^) | | | 128.2 (109.0, 158.3) | | | 136.7 (115.6, 161.8) | | | 133.8 (111.6, 155.0) | | | 0.21 | |  |  |
| **sPAP** (mmHg) | | | 45.4 (38.8, 53.9) | | | 49.1 (38.1, 57.3) | | | 46.6 (38.0, 56.7) | | | 0.48 | |  |  |
| **Global circumferential strain** () | | | -8.7 (-13.0, -6.3) | | | -8.9 (-13.6, -6.1) | | | -9.6 (-12.7, -6.7) | | | 0.65 | |  |  |
| **Global longitudinal strain** () | | | -10.3 (-14.3, -7.0) | | | -9.5 (-13.3, -6.5) | | | -11.5 (-14.9, -7.9) | | | 0.23 | |  |  |

**BMI** - Body Mass Index; **COPD** - Chronic Obstructive Pulmonary Disease; **MCV** - Mean Corpuscular Volume; **ACEi** - Angiotensin-Converting Enzyme Inhibitor; **ARB** - Angiotensin Receptor Blocker; **MRA** - Mineralocorticoid Receptor Antagonist; **RDW-CV** - Red Cell Distribution Width-CV; **AST** - Aspartate Aminotransferase; **HbA1c** - Hemoglobin A1c; **CKD-EPI eGFR** – CKD-EPI estimated Glomerular Filtration Rate; **BNP** - Brain Natriuretic Peptide; **LAVI** - Left Atrial Volume Index; **LVMI** - Left Ventricular Mass Index; **sPAP** - Systolic Pulmonary Arterial Pressure

**Table S2.** Adverse Clinical Outcomes by Discharge Ferritin Tertiles

| **Outcomes^1^** |  |  | |  | |
| --- | --- | --- | --- | --- | --- |
|  | **N (%)** | **Unadjusted** | *P-value* | **Adjusted^2^** | *P-value* |
| **HF Hospitalization or Cardiovascular Death** | |  |  |  |  |
| ***Tertile 1*** *[0.2-134.8 ng/mL]* | 37/132 (28.0) | *Reference* |  | *Reference* |  |
| ***Tertile 2*** *[135-286.4 ng/mL]* | 50/132 (37.9) | 1.44 (0.94-2.20) | 0.091 | 1.27 (0.76-2.12) | 0.4 |
| **Tertile 3** *[289.4 – 2545.2 ng/mL]* | 51/132 (38.6) | 1.54 (1.01-2.35) | **0.045** | 1.48 (0.80-2.73) | 0.2 |
| **HF Hospitalization** |  |  |  |  |  |
| ***Tertile 1*** *[0.2-134.8 ng/mL]* | 29/132 (22.0) | *Reference* |  | *Reference* |  |
| ***Tertile 2*** *[135-286.4 ng/mL]* | 40/132 (30.3) | 1.48 (0.92-2.38) | 0.1 | 1.42 (0.80-2.52) | 0.2 |
| **Tertile 3** *[289.4 – 2545.2 ng/mL]* | 35/132 (26.5) | 1.35 (0.83-2.21) | 0.2 | 1.53 (0.79-2.96) | 0.2 |
| **Cardiovascular Death** |  |  |  |  |  |
| ***Tertile 1*** *[0.2-134.8 ng/mL]* | 13/132 (9.9) | *Reference* |  | *Reference* |  |
| ***Tertile 2*** *[135-286.4 ng/mL]* | 22/132 (16.7) | 1.72 (0.86-3.40) | 0.1 | 1.19 (0.49-2.93) | 0.7 |
| **Tertile 3** *[289.4 – 2545.2 ng/mL]* | 25/132 (18.9) | 2.10 (1.07-4.09) | **0.030** | 1.83 (0.69-4.86) | 0.2 |
| **All-cause Hospitalization or Death** |  |  |  |  |  |
| ***Tertile 1*** *[0.2-134.8 ng/mL]* | 61/132 (46.2) | *Reference* |  | *Reference* |  |
| ***Tertile 2*** *[135-286.4 ng/mL]* | 68/132 (51.5) | 1.15 (0.81-1.62) | 0.4 | 1.24 (0.83-1.86) | 0.3 |
| **Tertile 3** *[289.4 – 2545.2 ng/mL]* | 73/132 (55.3) | 1.37 (0.97-1.93) | 0.068 | 1.52 (0.96-2.40) | 0.072 |
| **All-cause Hospitalization** |  |  |  |  |  |
| ***Tertile 1*** *[0.2-134.8 ng/mL]* | 49/132 (37.1) | *Reference* |  | *Reference* |  |
| ***Tertile 2*** *[135-286.4 ng/mL]* | 58/132 (43.9) | 1.22 (0.83-1.79) | 0.3 | 1.38 (0.85-2.24) | 0.2 |
| **Tertile 3** *[289.4 – 2545.2 ng/mL]* | 58/132 (43.9) | 1.36 (0.93-1.99) | 0.1 | 1.68 (0.98-2.88) | 0.058 |
| **All-cause Death** |  |  |  |  |  |
| ***Tertile 1*** *[0.2-134.8 ng/mL]* | 24/132 (18.1) | *Reference* |  | *Reference* |  |
| ***Tertile 2*** *[135-286.4 ng/mL]* | 33/132 (25.0) | 1.40 (0.83-2.37) | 0.2 | 1.07 (0.54-2.11) | 0.8 |
| **Tertile 3** *[289.4 – 2545.2 ng/mL]* | 42/132 (31.8) | 1.93 (1.17-3.19) | **0.010** | 1.31 (0.62-2.74) | 0.5 |

^1^ Hazard ratios (HR) are presented with their 95% confidence interval.

^2^ Adjusted for Sex, Age, Atrial fibrillation, Clinical infection and discharge NYHA class, Creatinine_log2_, BNP_log2_, Albumin, Haemoglobin, TSAT_log2_, Total cholesterol and CRP_log2_.

| **Outcomes^1^** | **ADMISSION** | | | |  | **DISCHARGE** | | | |
| --- | --- | --- | --- | --- | --- | --- | --- | --- | --- |
|  | **Unadjusted** | *P-value* | **Adjusted^2^** | *P-value* |  | **Unadjusted** | *P-value* | **Adjusted^2^** | *P-value* |
| **HF Hospitalization or Cardiovascular Death** | |  |  |  |  |  |  |  |  |
| ***<100*** *ng/mL* | *Reference* |  | *Reference* |  |  | *Reference* |  | *Reference* |  |
| ***≥100*** *&* ***299*** *ng/mL* | 1.40 (0.98-2.00) | 0.065 | 1.62 (1.03-2.55) | **0.038** |  | 1.74 (1.07-2.82) | **0.024** | 1.77 (0.97-3.23) | 0.061 |
| ***≥300*** *ng/mL* | 1.61 (1.09-2.37) | **0.016** | 1.56 (0.91-2.68) | 0.1 |  | 1.85 (1.12-3.06) | **0.017** | 2.01 (1.01-4.03) | **0.047** |
| **HF Hospitalization** |  |  |  |  |  |  |  |  |  |
| ***<100*** *ng/mL* | *Reference* |  | *Reference* |  |  | *Reference* |  | *Reference* |  |
| ***≥100*** *&* ***299*** *ng/mL* | 1.48 (0.97-2.24) | 0.068 | 1.46 (0.86-2.49) | 0.2 |  | 1.83 (1.06-3.15) | **0.030** | 2.13 (1.07-4.22) | **0.031** |
| ***≥300*** *ng/mL* | 1.36 (0.85-2.20) | 0.2 | 1.26 (0.66-2.40) | 0.5 |  | 1.62 (0.90-2.91) | 0.1 | 2.19 (0.98-4.88) | 0.055 |
| **Cardiovascular Death** |  |  |  |  |  |  |  |  |  |
| ***<100*** *ng/mL* | *Reference* |  | *Reference* |  |  | *Reference* |  | *Reference* |  |
| ***≥100*** *&* ***299*** *ng/mL* | 1.10 (0.65-1.87) | 0.7 | 1.54 (0.79-3.01) | 0.2 |  | 1.53 (0.72-3.28) | 0.3 | 1.11 (0.41-3.00) | 0.9 |
| ***≥300*** *ng/mL* | 1.58 (0.92-2.73) | 0.099 | 1.53 (0.68-3.47) | 0.3 |  | 2.19 (1.02-4.70) | **0.043** | 1.85 (0.60-5.71) | 0.3 |
| **All-cause Hospitalization or Death** |  |  |  |  |  |  |  |  |  |
| ***<100*** *ng/mL* | *Reference* |  | *Reference* |  |  | *Reference* |  | *Reference* |  |
| ***≥100*** *&* ***299*** *ng/mL* | 1.13 (0.85-1.52) | 0.4 | 1.26 (0.87-1.83) | 0.2 |  | 1.33 (0.91-1.94) | 0.1 | 1.47 (0.93-2.31) | 0.096 |
| ***≥300*** *ng/mL* | 1.44 (1.06-1.98) | **0.021** | 1.47 (0.95-2.30) | **0.085** |  | 1.57 (1.06-2.33) | **0.024** | 1.88 (1.10-3.19) | **0.020** |
| **All-cause Hospitalization** |  |  |  |  |  |  |  |  |  |
| ***<100*** *ng/mL* | *Reference* |  | *Reference* |  |  | *Reference* |  | *Reference* |  |
| ***≥100*** *&* ***299*** *ng/mL* | 1.21 (0.87-1.69) | 0.3 | 1.21 (0.79-1.87) | 0.4 |  | 1.36 (0.90-2.07) | 0.1 | 1.70 (1.03-2.81) | **0.039** |
| ***≥300*** *ng/mL* | 1.36 (0.94-1.96) | 0.1 | 1.27 (0.75-2.13) | 0.4 |  | 1.51 (0.97-2.35) | 0.062 | 2.21 (1.23-3.97) | **0.008** |
| **All-cause Death** |  |  |  |  |  |  |  |  |  |
| ***<100*** *ng/mL* | *Reference* |  | *Reference* |  |  | *Reference* |  | *Reference* |  |
| ***≥100*** *&* ***299*** *ng/mL* | 0.98 (0.66-1.47) | 0.9 | 1.09 (0.66-1.79) | 0.7 |  | 1.72 (0.95-3.13) | 0.076 | 1.25 (0.61-2.58) | 0.5 |
| ***≥300*** *ng/mL* | 1.30 (0.85-1.99) | 0.2 | 1.13 (0.61-2.10) | 0.7 |  | 2.29 (0.68-2.51) | **0.008** | 1.51 (0.66-3.48) | 0.3 |

**Table S3.** Adverse Clinical Outcomes by Guideline-defined ID Criteria Ferritin Cutoffs

^1^ Hazard ratios (HR) are presented with their 95% confidence interval.

^2^ Adjusted for Sex, Age, Atrial fibrillation, Clinical infection, NYHA class, Creatinine_log2_, BNP_log2_, Albumin, Haemoglobin, TSAT_log2_, Total cholesterol and CRP_log2_ at admission and discharge, respectively.

**Table S4.** Baseline Characteristics by Admission Iron Status and Guideline-defined Iron Deficiency Criteria.

| **Characteristic** | | | **NIS**  N = 128 | | | **DIU**  N = 180 | | **LIS**  N = 291 | | | **p-value** | | |  | **Guideline ID Criteria**  ***No***  N=283 | | | | | **Guideline ID Criteria**  ***Yes***  N=316 | | | | **p-value** | |  |
| --- | --- | --- | --- | --- | --- | --- | --- | --- | --- | --- | --- | --- | --- | --- | --- | --- | --- | --- | --- | --- | --- | --- | --- | --- | --- | --- |
| **Age** (years) | | | 80.0 (70.5, 84.5) | | | 77.0 (71.5, 84.0) | | 79.0 (72.0, 85.0) | | | 0.59 | | |  | 80.0 (71.0, 85.0) | | | | | 78.0 (72.0, 84.0) | | | | 0.17 | |  |
| **Male sex** | | | 69 (53.9) | | | 54 (30.0) | | 140(48.1) | | | **<0.001** | | |  | 149 (52.7) | | | | | 114 (36.1) | | | | **<0.001** | |  |
| **BMI** (Kg/m^2^) | | | 25.5 ± 5.1 | | | 25.7 ± 4.3 | | 25.1 ± 4.2 | | | 0.31 | | |  | 25.1 ± 4.3 | | | | | 25.6 ± 4.5 | | | | 0.26 | |  |
|  | | |  | | |  | |  | | |  | | |  |  | | | | |  | | | |  | |  |
| **PREVIOUS MEDICAL HISTORY** |  | | |  | | |  | |  | | |  | | | |  | |  | | |  | |  |  |  |  |
| **Dyslipidemia** | | | 59 (55.7) | | | 80 (54.4) | | 157 (62.5) | | | 0.22 | | |  | 133(56.4) | | | | | 163(60.8) | | | | 0.31 | |  |
| **Chronic Kidney Disease** | | | 27 (23.1) | | | 29 (17.0) | | 87 (30.6) | | | **0.004** | | |  | 78 (29.3) | | | | | 65 (21.2) | | | | **0.026** | |  |
| **Alcohol Consumption** | | | 60 (47.6) | | | 56 (31.5) | | 114 (39.7) | | | **0.016** | | |  | 124(44.6) | | | | | 106(33.9) | | | | **0.008** | |  |
| **Atrial Fibrillation** | | | 59 (46.1) | | | 95 (53.1) | | 116 (40.0) | | | **0.021** | | |  | 124(44.0) | | | | | 146(46.3) | | | | 0.56 | |  |
| **Anemia** | | | 52 (42.3) | | | 83 (48.3) | | 132 (46.2) | | | 0.59 | | |  | 124(45.1) | | | | | 143(46.7) | | | | 0.69 | |  |
| **Diabetes Mellitus** | | | 50 (39.7) | | | 69 (39.0) | | 118 (42.0) | | | 0.79 | | |  | 109(39.9) | | | | | 128(41.2) | | | | 0.76 | |  |
| **Myocardial Infarction** | | | 35 (27.8) | | | 51 (28.5) | | 95 (33.0) | | | 0.44 | | |  | 86 (30.7) | | | | | 95 (30.4) | | | | 0.92 | |  |
| **Hypertension** | | | 91 (76.5) | | | 132(75.0) | | 210 (75.3) | | | 0.96 | | |  | 196(73.7) | | | | | 237(76.9) | | | | 0.37 | |  |
| **COPD** | | | 25 (23.6) | | | 27 (18.6) | | 51 (21.4) | | | 0.62 | | |  | 50 (21.8) | | | | | 53 (20.4) | | | | 0.69 | |  |
|  | | |  | | |  | |  | | |  | | |  |  | | | | |  | | | |  | |  |
| **PREVIOUS MEDICATION** | | |  | | |  | |  | | |  | | |  |  | | | | |  | | | |  | |  |
| **Beta-blocker** | | | 63 (51.2) | | | 71 (39.4) | | 155 (53.8) | | | **0.009** | | |  | 140(50.7) | | | | | 149(47.3) | | | | 0.41 | |  |
| **ACEi or ARB** | | | 79 (64.2) | | | 103(57.2) | | 186 (64.6) | | | 0.25 | | |  | 176(63.8) | | | | | 192(61.0) | | | | 0.48 | |  |
| **MRA** | | | 22 (17.9) | | | 23 (12.8) | | 41 (14.2) | | | 0.45 | | |  | 43 (15.6) | | | | | 43 (13.7) | | | | 0.51 | |  |
| **Loop diuretics** | | | 92 (74.2) | | | 124 (68.9) | | 215 (74.1) | | | 0.42 | | |  | 200(71.9) | | | | | 231(73.1) | | | | 0.75 | |  |
| **Statins** | | | 57 (46.3) | | | 86 (47.8) | | 146 (50.7) | | | 0.68 | | |  | 127(46.0) | | | | | 162(51.4) | | | | 0.19 | |  |
|  | | |  | | |  | |  | | |  | | |  |  | | | | |  | | | |  | |  |
| **CLINICAL PRESENTATION AND HOSPITAL STAY** | | |  | | |  | |  | | |  | | |  |  | | | | |  | | | |  | |  |
| **Systolic blood pressure** (mmHg) | | | 131.0 (113.0, 153.0) | | | 133.5 (114.5, 155.5) | | 128.0 (112.0, 150.0) | | | 0.43 | | |  | 129.0 (111.0, 150.0) | | | | | 131.0 (116.0, 151.0) | | | | 0.15 | |  |
| **Diastolic blood pressure** (mmHg) | | | 71.5 (63.0, 90.0) | | | 76.0 (66.0, 90.0) | | 73.0 (60.0, 85.0) | | | 0.10 | | |  | 70.0 (60.0, 87.0) | | | | | 76.0 (66.0, 89.0) | | | | **0.003** | |  |
| **Heart rate** (bpm) | | | 85.0 (68.0, 102.0) | | | 87.0 (74.5, 102.0) | | 86.0 (72.0, 100.0) | | | 0.42 | | |  | 87.0 (70.0, 102.0) | | | | | 85.0 (74.0, 100.0) | | | | 0.96 | |  |
| **Clinical infection** | | | 41 (33.3) | | | 62 (35.2) | | 162 (57.9) | | | **<0.001** | | |  | 131(48.5) | | | | | 134(43.4) | | | | 0.21 | |  |
| **Length of Stay** (days) | | | 8.0 (5.0, 14.0) | | | 8.0 (6.0, 12.0) | | 8.0 (6.0, 11.0) | | | 0.63 | | |  | 8.0 (6.0 ,13.0) | | | | | 8.0 (5.0 ,11.0) | | | | 0.12 | |  |
|  | | |  | | |  | |  | | |  | | |  |  | | | | |  | | | |  | |  |
| **ADMISSION ANALYTICAL EVALUATION** |  | | |  | | |  | |  | | |  | | | |  | |  | | |  | |  |  |  |  |
| **Hemoglobin** (g/dL) | | | 12.0 (10.3, 14.0) | | | 11.7 (10.3, 13.3) | | 11.4 (10.1, 12.9) | | | 0.07 | | |  | 11.7 (10.1, 13.5) | | | | | 11.6 (10.3, 13.0) | | | | 0.39 | |  |
| **MCV** (fL) | | | 92.4 (88.4, 96.4) | | | 87.6 (81.8, 92.3) | | 91.4 (87.1, 95.1) | | | **<0.001** | | |  | 92.0 (88.0, 95.8) | | | | | 89.4 (83.8, 93.2) | | | | **<0.001** | |  |
| **RDW-CV** () | | | 14.8 (13.8, 16.1) | | | 15.5 (14.3, 17.1) | | 14.8 (13.9, 16.2) | | | **<0.001** | | |  | 14.7 (13.9, 16.2) | | | | | 15.2 (14.2, 16.7) | | | | **0.004** | |  |
| **AST** (U/L) | | | 25.0 (20.0, 36.0) | | | 27.0 (21.0, 34.0) | | 28.0 (20.0, 40.0) | | | 0.58 | | |  | 28.0 (21.0, 41.0) | | | | | 27.0 (20.0, 35.0) | | | | 0.09 | |  |
| **ALT** (U/L) | | | 21.0 (16.0, 32.0) | | | 21.0 (14.0, 32.0) | | 21.0 (14.0, 39.0) | | | 0.71 | | |  | 21.0 (16.0, 38.0) | | | | | 20.0 (14.0, 33.0) | | | | 0.06 | |  |
| **Albumin** (g/L) | | | 36.1 (31.7, 39.4) | | | 35.7 (32.6, 38.6) | | 35.0 (31.8, 38.0) | | | 0.20 | | |  | 35.5 (31.7, 38.5) | | | | | 35.5 (32.4, 38.2) | | | | 0.58 | |  |
| **Total cholesterol** (mg/dL) | | | 158.0 (132.0, 189.0) | | | 146.0 (120.0, 182.0) | | 146.0 (122.0, 183.0) | | | 0.10 | | |  | 154.0 (126.0, 189.0) | | | | | 146.0 (119.0, 181.0) | | | | 0.07 | |  |
| **HbA1c** (%) | | | 6.1 (5.6, 6.8) | | | 6.3 (5.8, 7.1) | | 6.1 (5.7, 6.8) | | | 0.12 | | |  | 6.1 (5.7, 6.8) | | | | | 6.3 (5.8, 7.0) | | | | 0.14 | |  |
| **CKD-EPI eGFR** (mL/min/1.73m^2^) | | | 42.6 (28.7, 59.3) | | | 43.1 (30.6, 56.1) | | 40.5 (26.7, 56.1) | | | 0.35 | | |  | 41.8 (27.3, 56.9) | | | | | 42.3 (30.1, 56.3) | | | | 0.70 | |  |
| **Urea** (mg/dL) | | | 64.0 (46.0, 102.0) | | | 65.0 (44.0, 93.0) | | 71.0 (51.0, 103.0) | | | 0.11 | | |  | 71.0 (51.0, 106.0) | | | | | 65.0 (46.0, 91.0) | | | | **0.029** | |  |
| **Iron** (μg/mL) | | | 75.0 (38.5, 95.5) | | | 31.0 (21.5, 46.0) | | 35.0 (24.0, 47.0) | | | **<0.001** | | |  | 42.0 (29.0, 71.0) | | | | | 34.8 (23.0, 48.3) | | | | **<0.001** | |  |
| **Transferrin** (mg/dL) | | | 215.0 (179.0, 254.0) | | | 292.0 (253.8, 331.0) | | 221.0 (185.0, 261.5) | | | **<0.001** | | |  | 214.0 (178.0, 252.8) | | | | | 265.5 (227.3, 312.0) | | | | **<0.001** | |  |
| **Transferrin Saturation** () | | | 26.0 (22.6, 34.1) | | | 7.8 (5.4, 12.0) | | 11.4 (7.6, 15.1) | | | **<0.001** | | |  | 16.5 (9.9, 24.2) | | | | | 9.5 (6.1, 13.7) | | | | **<0.001** | |  |
| **C-reactive protein** (mg/L) | | | 13.2 (5.7, 39.8) | | | 13.3 (6.9, 25.6) | | 36.0 (14.0, 88.5) | | | **<0.001** | | |  | 25.1 (10.2, 72.2) | | | | | 17.9 (9.0, 51.1) | | | | **0.012** | |  |
| **Troponin I** (ng/mL) | | | 0.1 (0.0, 0.1) | | | 0.1 (0.0, 0.2) | | 0.1 (0.0, 0.1) | | | 0.57 | | |  | 0.1 (0.0, 0.1) | | | | | 0.1 (0.0, 0.1) | | | | 0.46 | |  |
| **BNP** (pg/mL) | | | 1091.5 (639.1, 2435.4) | | | 1293.2 (757.0, 2714.9) | | 1370.5 (680.9, 2607.1) | | | 0.25 | | |  | 1354.8 (672.0, 2617.9) | | | | | 1293.0 (711.9, 2563.6) | | | | 0.91 | |  |
|  | |  | | |  | | |  | |  | | |  | | | |  | |  | | |  | | |  | |
| **ECHOCARDIOGRAPHIC EVALUATION** | | |  | | |  | |  | | |  | | |  |  | | | | |  | | | |  | |  |
| **Ejection Fraction** (%) | | | 40.0 (25.5, 56.0) | | | 42.0 (23.0, 57.0) | | 40.0 (26.0, 56.5) | | | 0.84 | | |  | 39.0 (26.0, 56.5) | | | | | 43.0 (23.0, 57.0) | | | | 0.98 | |  |
| **LAVI** (mL/m^2^) | | | 48.1 (38.6, 62.8) | | | 51.9 (41.0, 66.0) | | 47.2 (37.9, 60.4) | | | 0.07 | | |  | 46.7 (37.3, 60.8) | | | | | 50.2 (40.1, 64.1) | | | | **0.025** | |  |
| **LVMI** (g/m^2^) | | | 132.5 (111.8, 160.2) | | | 128.5 (108.9, 159.1) | | 133.4 (111.9, 156.2) | | | 0.92 | | |  | 132.7 (112.3, 155.2) | | | | | 132.1 (109.0, 159.2) | | | | 0.77 | |  |
| **sPAP** (mmHg) | | | 47.1 (37.9, 60.9) | | | 46.2 (38.6, 55.0) | | 47.2 (38.4, 57.2) | | | 0.43 | | |  | 46.8 (38.6, 59.4) | | | | | 47.0 (38.1, 55.7) | | | | 0.40 | |  |
| **Global circumferential strain** (%) | | | -9.2 (-13.0, -6.2) | | | -8.2 (-11.5, -6.1) | | -9.6 (-13.4, -6.5) | | | 0.36 | | |  | -9.0 (-12.8, -6.4) | | | | | -9.0 (-12.9, -6.3) | | | | 0.79 | |  |
| **Global longitudinal strain** (%) | | | -10.1 (-14.5, -6.5) | | | -9.6 (-14.0, -7.4) | | -10.3 (-15.5, -6.6) | | | 0.93 | | |  | -10.1 (-14.9, -6.4) | | | | | -10.2 (-15.2, -7.6) | | | | 0.23 | |  |

**ACEi** - Angiotensin-converting enzyme inhibitor, **ARB** - Angiotensin receptor blocker, **AST** - Aspartate aminotransferase, **BMI** - Body mass index, **BNP** - Brain natriuretic peptide, **CKD-EPI eGFR** - CKD-EPI estimated glomerular filtration rate, **COPD** - Chronic obstructive pulmonary disease, **DIU** - Defective iron utilization, **HbA1c** - Hemoglobin A1c, **LAVI** - Left atrial volume index, **LIS** - Low iron storage, **LVMI** - Left ventricular mass index, **MCV** - Mean corpuscular volume, **MRA** - Mineralocorticoid receptor antagonist, **NIS** - Normal iron status, **RDW-CV** - Red cell distribution width-CV, and **sPAP** - Systolic pulmonary arterial pressure**.**

**Table S5.** Baseline Characteristics by Discharge Iron Status and Guideline-defined Iron Deficiency Criteria

| **Characteristic** | | | **NIS**  N = 162 | | | **DIU**  N = 102 | | **LIS**  N = 187 | | | **p-value** | | |  | **Guideline ID Criteria**  ***No***  N = 259 | | | | | **Guideline ID Criteria**  ***Yes***  N = 192 | | | | **p-value** | |  |
| --- | --- | --- | --- | --- | --- | --- | --- | --- | --- | --- | --- | --- | --- | --- | --- | --- | --- | --- | --- | --- | --- | --- | --- | --- | --- | --- |
| **Age** (years) | | | 78.5 (70.0, 85.0) | | | 75.0 (70.0, 83.0) | | 78.0 (71.0, 84.0) | | | 0.62 | | |  | 79.0 (70.0, 84.0) | | | | | 77.0 (71.5, 83.0) | | | | 0.42 | |  |
| **Male sex** | | | 86 (53.1) | | | 29 (28.4) | | 89 (47.6) | | | **<0.001** | | |  | 129 (49.8) | | | | | 75 (39.1) | | | | **0.023** | |  |
| **BMI** (Kg/m^2^) | | | 24.7 ± 4.4 | | | 25.9 ± 4.5 | | 25.4 ± 4.3 | | | 0.10 | | |  | 24.9 ± 4.3 | | | | | 25.8 ± 4.5 | | | | **0.028** | |  |
|  | | |  | | |  | |  | | |  | | |  |  | | | | |  | | | |  | |  |
| **PREVIOUS MEDICAL HISTORY** |  | | |  | | |  | |  | | |  | | | |  | |  | | |  | |  |  |  |  |
| **Dyslipidemia** | | | 73 (53.7) | | | 53 (60.2) | | 101(59.8) | | | 0.49 | | |  | 123 (54.9) | | | | | 104 (61.5) | | | | 0.19 | |  |
| **Chronic Kidney Disease** | | | 38 (24.7) | | | 18 (18.2) | | 45 (24.9) | | | 0.39 | | |  | 64 (25.8) | | | | | 37 (19.9) | | | | 0.15 | |  |
| **Alcohol Consumption** | | | 75 (46.6) | | | 32 (31.7) | | 74 (40.0) | | | 0.06 | | |  | 118 (45.7) | | | | | 63 (33.3) | | | | **0.008** | |  |
| **Atrial Fibrillation** | | | 69 (42.6) | | | 53 (52.5) | | 75 (40.1) | | | 0.12 | | |  | 108 (41.7) | | | | | 89 (46.6) | | | | 0.3 | |  |
| **Anemia** | | | 67 (42.9) | | | 48 (48.0) | | 99 (54.1) | | | 0.12 | | |  | 123 (48.6) | | | | | 91 (48.9) | | | | 0.95 | |  |
| **Diabetes Mellitus** | | | 52 (32.3) | | | 46 (45.5) | | 85 (46.2) | | | **0.019** | | |  | 94 (36.7) | | | | | 89 (46.8) | | | | **0.032** | |  |
| **Myocardial Infarction** | | | 53 (33.1) | | | 29 (28.7) | | 58 (31.2) | | | 0.75 | | |  | 82 (31.9) | | | | | 58 (30.5) | | | | 0.76 | |  |
| **Hypertension** | | | 112(72.3) | | | 81 (79.4) | | 138(75.0) | | | 0.43 | | |  | 179 (71.3) | | | | | 152 (80.0) | | | | **0.037** | |  |
| **COPD** | | | 29 (21.8) | | | 15 (17.4) | | 35 (21.2) | | | 0.71 | | |  | 46 (20.7) | | | | | 33 (20.4) | | | | 0.93 | |  |
|  | | |  | | |  | |  | | |  | | |  |  | | | | |  | | | |  | |  |
| **PREVIOUS MEDICATION** | | |  | | |  | |  | | |  | | |  |  | | | | |  | | | |  | |  |
| **Beta-blocker** | | | 84 (52.5) | | | 43 (42.2) | | 95 (51.9) | | | 0.20 | | |  | 134 (52.5) | | | | | 88 (46.3) | | | | 0.19 | |  |
| **ACEi or ARB** | | | 103(64.4) | | | 59 (57.8) | | 117(63.9) | | | 0.51 | | |  | 165 (64.7) | | | | | 114 (60.0) | | | | 0.31 | |  |
| **MRA** | | | 27 (16.9) | | | 15 (14.7) | | 27 (14.8) | | | 0.84 | | |  | 43 (16.9) | | | | | 26 (13.7) | | | | 0.36 | |  |
| **Loop diuretics** | | | 117(72.7) | | | 65 (63.7) | | 144(77.8) | | | **0.037** | | |  | 189 (73.5) | | | | | 137 (71.7) | | | | 0.67 | |  |
| **Statins** | | | 73 (45.6) | | | 55 (53.9) | | 89 (48.6) | | | 0.42 | | |  | 120 (47.1) | | | | | 97 (51.1) | | | | 0.4 | |  |
|  | | |  | | |  | |  | | |  | | |  |  | | | | |  | | | |  | |  |
| **CLINICAL PRESENTATION AND HOSPITAL STAY** | | |  | | |  | |  | | |  | | |  |  | | | | |  | | | |  | |  |
| **Systolic blood pressure** (mmHg) | | | 112.0 (102.0, 124.0) | | | 114.0 (102.0, 130.0) | | 110.0 (100.0, 125.0) | | | 0.30 | | |  | 110.0 (100.0, 124.0) | | | | | 114.0 (101.0, 130.0) | | | | **0.03** | |  |
| **Diastolic blood pressure** (mmHg) | | | 64.0 (57.0, 70.0) | | | 64.0 (54.0, 72.0) | | 64.0 (56.0, 73.0) | | | 0.98 | | |  | 65.0 (57.0, 71.0) | | | | | 64.0 (54.0, 73.0) | | | | 0.55 | |  |
| **Heart rate** (bpm) | | | 75.0 (66.0, 85.0) | | | 71.0 (64.5, 80.0) | | 72.0 (66.0, 81.0) | | | 0.26 | | |  | 74.5 (67.0, 85.0) | | | | | 72.0 (65.0, 80.0) | | | | **0.05** | |  |
| **Clinical infection** | | | 83 (51.9) | | | 31 (30.7) | | 91 (50.0) | | | **0.002** | | |  | 138(54.3) | | | | | 67 (35.4) | | | | **0.001** | |  |
| **Length of Stay** (days) | | | 8.0 (6.0, 13.0) | | | 7.0 (5.0, 10.0) | | 8.0 (5.0, 11.0) | | | **0.046** | | |  | 8.0 (6.0, 13.0) | | | | | 7.0 (5.0, 11.0) | | | | **0.012** | |  |
|  | | |  | | |  | |  | | |  | | |  |  | | | | |  | | | |  | |  |
| **ADMISSION ANALYTICAL EVALUATION** |  | | |  | | |  | |  | | |  | | | |  | |  | | |  | |  |  |  |  |
| **Hemoglobin** (g/dL) | | | 12.4 (11.1, 13.8) | | | 11.5 (10.5, 12.3) | | 11.3 (10.2, 12.7) | | | **<0.001** | | |  | 12.0 (10.7, 13.5) | | | | | 11.5 (10.4, 12.4) | | | | **0.01** | |  |
| **MCV** (fL) | | | 92.0 (88.8, 94.8) | | | 85.5 (82.0, 90.0) | | 90.1 (85.7, 94.3) | | | **<0.001** | | |  | 91.6 (87.9, 94.5) | | | | | 87.8 (83.4, 92.2) | | | | **0.001** | |  |
| **AST** (U/L) | | | 26.0 (21.0, 38.0) | | | 24.0 (16.5, 32.0) | | 26.0 (20.0, 35.0) | | | **0.033** | | |  | 27.0 (21.0, 37.0) | | | | | 24.0 (17.0, 32.0) | | | | **0.004** | |  |
| **Albumin** (g/L) | | | 35.5 (31.6, 38.1) | | | 35.4 (32.0, 38.7) | | 34.8 (31.8, 37.5) | | | 0.42 | | |  | 34.9 (31.5, 37.6) | | | | | 35.1 (32.1, 38.2) | | | | 0.27 | |  |
| **Total cholesterol** (mg/dL) | | | 157.0 (132.0, 188.0) | | | 141.5 (115.0, 170.0) | | 144.0 (121.0, 172.0) | | | **0.003** | | |  | 152.0 (127.0, 183.0) | | | | | 143.0 (118.5, 171.5) | | | | **0.018** | |  |
| **HbA1c** () | | | 6.0 (5.6, 6.7) | | | 6.5 (5.9, 7.7) | | 6.3 (5.8, 7.3) | | | **<0.001** | | |  | 6.1 (5.7, 6.9) | | | | | 6.3 (5.9 7.1) | | | | **0.011** | |  |
| **CKD-EPI eGFR** (mL/min/1.73m^2^) | | | 44.6 (34.3, 60.9) | | | 47.3 (34.7, 59.0) | | 41.5 (30.6, 54.8) | | | 0.17 | | |  | 44.4 (32.7, 63.6) | | | | | 42.7 (31.3, 54.7) | | | | 0.17 | |  |
| **Urea** (mg/dL) | | | 73.0 (51.0, 103.0) | | | 59.0 (46.0, 92.0) | | 74.0 (53.0, 102.5) | | | 0.10 | | |  | 71.5 (51.0, 103.0) | | | | | 71.5 (51.0, 97.0) | | | | 0.68 | |  |
| **Iron** (mg/mL) | | | 91.0 (74.0, 113.0) | | | 42.8 (32.0, 56.5) | | 39.5 (30.0, 51.0) | | | **<0.001** | | |  | 68.3 (42.3, 97.0) | | | | | 42.3 (32.0, 56.0) | | | | **0.001** | |  |
| **Transferrin** (mg/dL) | | | 224.5 (193.0, 260.0) | | | 293.5 (248.0, 341.0) | | 232.0 (195.5, 276.0) | | | **<0.001** | | |  | 221.0 (187.0, 260.0) | | | | | 273.3 (228.3, 321.0) | | | | **0.001** | |  |
| **Transferrin Saturation** () | | | 27.8 (22.9, 34.0) | | | 10.9 (8.3, 14.0) | | 12.3 (9.0, 15.8) | | | **<0.001** | | |  | 21.8 (14.9, 29.3) | | | | | 11.5 (8.8, 14.9) | | | | **0.001** | |  |
| **C-reactive protein** (mg/L) | | | 10.8 (5.1, 18.5) | | | 9.4 (4.1, 17.8) | | 19.3 (9.1, 40.0) | | | **<0.001** | | |  | 13.2 (6.3, 25.9) | | | | | 10.6 (5.9, 24.9) | | | | 0.26 | |  |
| **Troponin I** (ng/mL) | | | 0.0 (0.0, 0.1) | | | 0.0 (0.0, 0.1) | | 0.0 (0.0, 0.1 ) | | | 0.75 | | |  | 0.0 (0.0, 0.1) | | | | | 0.0 (0.0, 0.1) | | | | 0.75 | |  |
| **BNP** (pg/mL) | | | 702.8 (302.3, 1293.9) | | | 704.5 (303.1, 1582.9) | | 842.3 (350.0, 1447.9) | | | 0.22 | | |  | 754.5 (319.0, 1387.3) | | | | | 788.0 (324.9, 1556.6) | | | | 0.57 | |  |
|  | |  | | |  | | |  | |  | | |  | | | |  | |  | | |  | | |  | |
| **ECHOCARDIOGRAPHIC EVALUATION** | | |  | | |  | |  | | |  | | |  |  | | | | |  | | | |  | |  |
| **Ejection Fraction** () | | | 38.8 (23.5, 57.5) | | | 43.0 (22.5, 57.0) | | 36.5 (24.0, 55.0) | | | 0.69 | | |  | 38.0 (23.5, 56.5) | | | | | 41.0 (23.0, 55.0) | | | | 0.68 | |  |
| **LAVI** (mL/m^2^) | | | 45.7 (36.7, 60.4) | | | 51.7 (38.6, 62.3) | | 50.0 (41.2, 64.9) | | | 0.08 | | |  | 47.7 (37.9, 62.6) | | | | | 50.7 (39.6, 62.6) | | | | 0.48 | |  |
| **LVMI** (g/m^2^) | | | 136.6 (112.3, 156.8) | | | 128.7 (108.7, 161.4) | | 129.2 (111.3, 157.0) | | | 0.75 | | |  | 135.3 (111.2, 156.2) | | | | | 129.2 (111.0, 161.8) | | | | 0.76 | |  |
| **sPAP** (mmHg) | | | 46.7 (37.1, 56.4) | | | 45.5 (38.9, 52.9) | | 48.6 (39.6, 58.2) | | | 0.25 | | |  | 47.6 (38.9, 57.2) | | | | | 46.1 (38.9, 55.7) | | | | 0.45 | |  |
| **Global circumferential strain** (%) | | | -9.2 (-12.8, -6.2) | | | -8.4 (-11.6, -6.0) | | -9.2 (-12.6, -6.4) | | | 0.72 | | |  | -9.1 (-12.7, -6.2) | | | | | -8.9 (-12.6, -6.3) | | | | 0.87 | |  |
| **Global longitudinal strain** (%) | | | -10.2 (-14.3, -6.5) | | | -10.0 (-14.0, -6.9) | | -9.8 (-15.2, -7.2) | | | 0.72 | | |  | -10.4 (-14.9, -7.0) | | | | | -9.6 (-14.0, -6.9) | | | | 0.33 | |  |

**ACEi** - Angiotensin-converting enzyme inhibitor, **ARB** - Angiotensin receptor blocker, **AST** - Aspartate aminotransferase, **BMI** - Body mass index, **BNP** - Brain natriuretic peptide, **CKD-EPI eGFR** - CKD-EPI estimated glomerular filtration rate, **COPD** - Chronic obstructive pulmonary disease, **DIU** - Defective iron utilization, **HbA1c** - Hemoglobin A1c, **LAVI** - Left atrial volume index, **LIS** - Low iron storage, **LVMI** - Left ventricular mass index, **MCV** - Mean corpuscular volume, **MRA** - Mineralocorticoid receptor antagonist, **NIS** - Normal iron status, **RDW-CV** - Red cell distribution width-CV, and **sPAP** - Systolic pulmonary arterial pressure**.**

**Table S6.** Primary Composite Endpoint by Discharge Serum Iron, Transferrin Saturation, and Iron Status.

| **PRIMARY COMPOSITE ENDPOINT^1^** | |  |  |  |  |  |
| --- | --- | --- | --- | --- | --- | --- |
|  |  |  |  |  |  |  |
| **DISCHARGE** | **N (%)** | **Unadjusted** | *P-value* | **Adjusted** | *P-value* |  |
| **SERUM IRON^2^** |  |  |  |  |  |  |
| ***Tertile 1*** *[6 – 41 mg/mL]* | 53/131 (40.6) | 1.31 (0.87-1.97) | 0.3 | 0.75 (0.44-1.25) | 0.3 |  |
| ***Tertile 2*** *[42 – 68 mg/mL]* | 36/124 (29.0) | 0.88 (0.57-1.39) | 0.9 | 0.59 (0.36-0.98) | **0.043** |  |
| ***Tertile 3*** *[68 – 228 mg/mL]* | 41/126 (32.5) | *Reference* |  | *Reference* |  |  |
| **TRANSFERRIN SATURATION^2^** | |  |  |  |  |  |
| ***Tertile 1*** *[1.73-11.76 %]* | 50/127 (39.3) | 0.99 (0.67-1.46) | 0.9 | 0.66 (0.41-1.05) | 0.084 |  |
| ***Tertile 2*** *[11.77-20.23 %]* | 30/127 (23.6) | 0.54 (0.34-0.86) | **0.009** | 0.39 (0.23-0.65) | **<0.001** |  |
| ***Tertile 3*** *[20.27-80.14 %]* | 50/127 (39.3) | *Reference* |  | *Reference* |  |  |
| **IRON STATUS^2^** |  |  |  |  |  |  |
| ***Normal Iron Status*** | 58/141 (41.1) | *Reference* |  | *Reference* |  |  |
| ***Deficient Iron Utilization*** | 25/102 (24.5) | 0.52 (0.32-0.83) | **0.006** | 0.41 (0.23-0.71) | **0.002** |  |
| ***Low Iron Storage*** | 55/153 (36.0) | 0.84 (0.58-1.21) | 0.4 | 0.59 (0.39-0.92) | **0.018** |  |
| **ANEMIA^3^** |  |  |  |  |  |  |
| ***None***  ***Mild***  ***Moderate/Severe*** | 44/153 (28.8)  27/77 (35.1)  67/213 (40.4) | *Reference*  1.22 (0.75-1.97)  1.48 (1.01-2.16) | 0.4  **0.043** | *Reference*  1.04 (0.59-1.86)  1.11 (0.68-1.81) | 0.9  0.7 |  |
| **GUIDELINE ID CRITERIA^2^** |  |  |  |  |  |  |
| ***Yes*** | 59/192 (30.7) | *Reference* |  | *Reference* |  |  |
| ***No*** | 79/1204 (38.7) | 1.37 (0.98-1.92) | 0.067 | 1.63 (1.09-2.42) | **0.017** |  |

^1^ Hazard ratios (HR) are presented with their 95% confidence interval.

^2^ Adjusted for Adjusted for Sex, Age, Atrial fibrillation, NYHA class, Creatinine _log2_, BNP_log2_, Albumin, Haemoglobin, Total cholesterol, CRP_log2_ and Clinical infection.

_3_ . Adjusted for Sex, Age, Atrial fibrillation, NYHA class, Creatinine _log2_, BNP_log2_, Albumin, TSAT_log2_, Total cholesterol, CRP_log2_ and Clinical infection.

**Table S7.** Variation Between Admission and Discharge Percentages of Patients Meeting Established Cutoffs for Ferritin, Transferrin Saturation, Iron, and Anemia

|  | **Admission^1^** | **Discharge** | **Variation** |
| --- | --- | --- | --- |
| **FERRITIN** |  |  |  |
| **<15 ng/mL** (*WHO criteria*) | 10 (2.5) | 4 (1.0) | -60.0% |
| **<30 ng/mL** (*Standard lab criteria*) | 36 (7.6) | 13 (3.3) | -56.5% |
| **<100 ng/mL** (*Guideline ID criteria*) | 130 (32.8) | 91 (23.0) | -29.8% |
| **100-299 ng/mL** (*Guideline ID criteria*) | 166 (41.9) | 177 (44.7) | +6.7% |
| **TRANSFERRIN SATURATION** |  |  |  |
| **<16** **%** *(WHO criterion)* | 260 (69.3) | 204 (53.5) | -22.8% |
| **<20** **%** (*Standard lab / Guideline ID criteria*) | 308 (82.1) | 255 (66.9) | -18.5% |
| **SERUM IRON^2^** |  |  |  |
| **<65 μg/dL** (*WHO criterion – Male*) | 126 (70.4) | 97 (54.2) | -23.0 |
| **<50 μg/dL** (*WHO criterion – Female*) | 144 (66.4) | 109 (50.2) | -24.3 |
| **≤73 μg/dL** / **≤13 µmol/L** ^3^ | 322 (85.6) | 270 (70.9) | -17.2 |
| **ANEMIA^4^** |  |  |  |
| *No* | 136 (34.3) | 153 (38.6) | +12.5 |
| *Mild* | 77 (19.4) | 77 (19.4) | 0.0 |
| *Moderate/Severe* | 183 (46.2) | 166 (41.9) | -9.3 |

1 – For the purpose of comparative analysis, only patients with available discharge ferritin levels (N=396) were included in this analysis.

2 - Serum iron conversion factor from mg/mL to μmol/μL - divide by 5.59

3 – Serum iron cutoff based on the Ref ^12^

4 – Anemia cutoffs based on the Ref ^11^

**Figure S1.**

**B**

**A**

**Figure S2.**

**B**

**A**
